# Extranodal Natural Killer/T-cell Lymphoma Isolated to the Leg: A Case Report

**DOI:** 10.7759/cureus.40011

**Published:** 2023-06-05

**Authors:** Sharon Pan, Nada Mohamed, Saadeddine Saad, Palak Parekh

**Affiliations:** 1 Dermatology, Baylor Scott & White Medical Center - Temple, Temple, USA; 2 Pathology, Baylor Scott & White Medical Center - Temple, Temple, USA

**Keywords:** general dermatology, dermatopathology, skin disease/ dermatology, onco dermatology, extranodal natural killer/t-cell lymphoma

## Abstract

Extranodal natural killer/T-cell lymphoma (ENKTL) is a subtype of non-Hodgkin's lymphoma, and it is exceedingly rare in North America. The "extranasal" subtype of ENKTL frequently involves the skin and typically has an aggressive course with no current standard of treatment available. In this report, we present a case of cutaneous ENKTL in an otherwise healthy middle-aged male.

## Introduction

Extranodal natural killer/T-cell lymphoma (ENKTL) is a subtype of non-Hodgkin's lymphoma, and it is further classified into nasal (primary upper nasopharynx involvement) and extranasal (primary peripheral involvement outside of the nasal region) types. There is a scarcity of data on this type of lymphoma, and it is exceedingly rare in North America [1,2]. The non-nasal cases are even more uncommon, with the skin being one of the more frequent sites of involvement [2]. The disease has an aggressive course, and there is no standard of care currently [2]. We report a case of cutaneous ENKTL in an otherwise healthy middle-aged male.

The patient provided verbal consent for the use of photos and publication of this report.

## Case presentation

**Figure 1:**
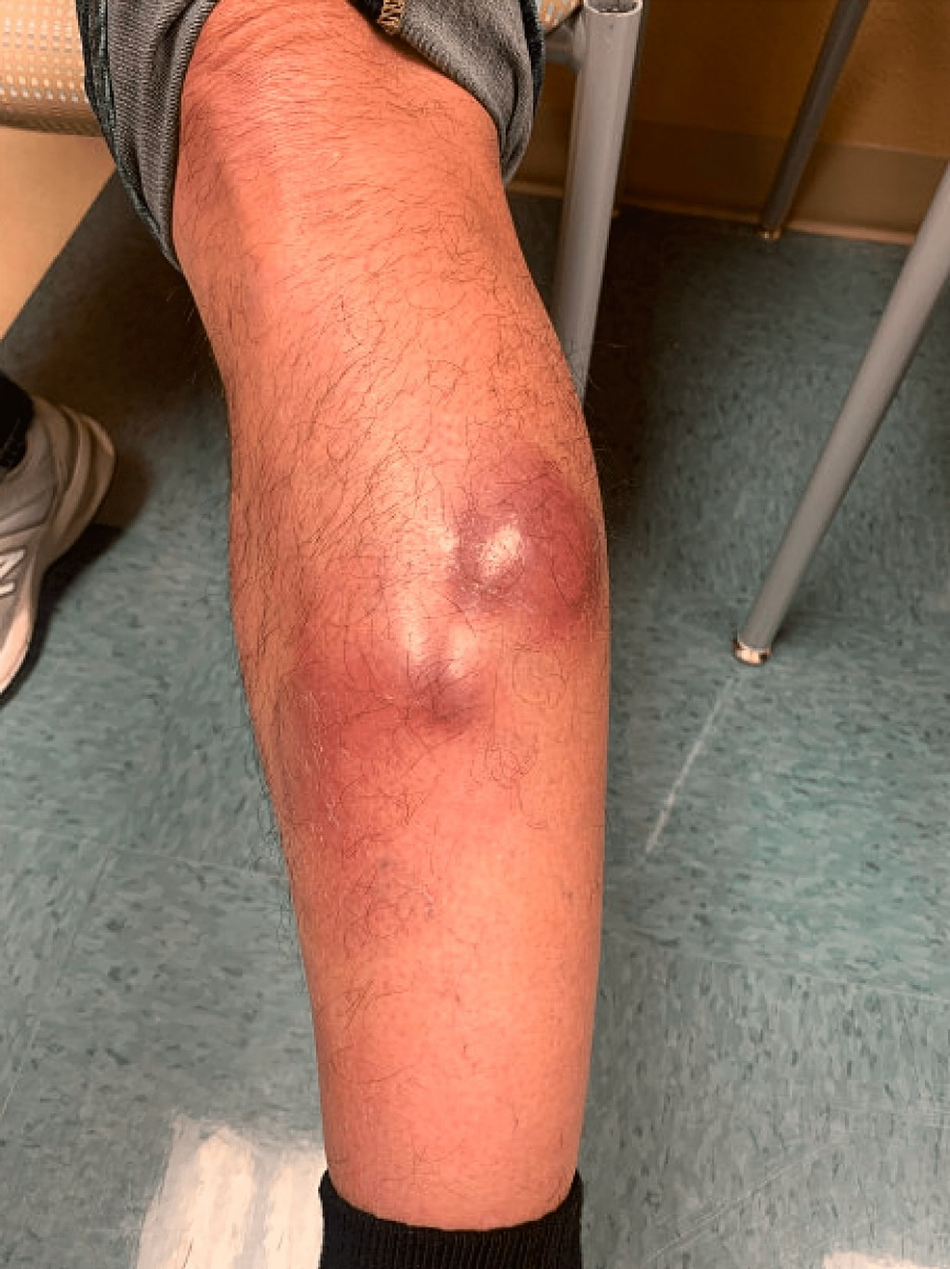
Initial presentation of extranodal natural killer/T-cell lymphoma (ENKTL) with erythematous to violaceous nodules involving the left lower leg

A 67-year-old male with no significant past medical history presented to the Dermatology Department at the end of 2021 with skin lesions isolated to the left lower leg for a year. The lesions had failed to resolve with cephalexin and amoxicillin-clavulanate. Physical exam revealed indurated, approximately three erythematous nodules, slightly tender to palpation, on the left lower pretibial leg (Figure 1) with sparing of the right leg. The largest lesion measured approximately 6 cm in the longest diameter. There was no history of similar lesions elsewhere and a thorough dermatological exam did not reveal any other skin lesions. No palpable lymph nodes were appreciated.

**Figure 2:**
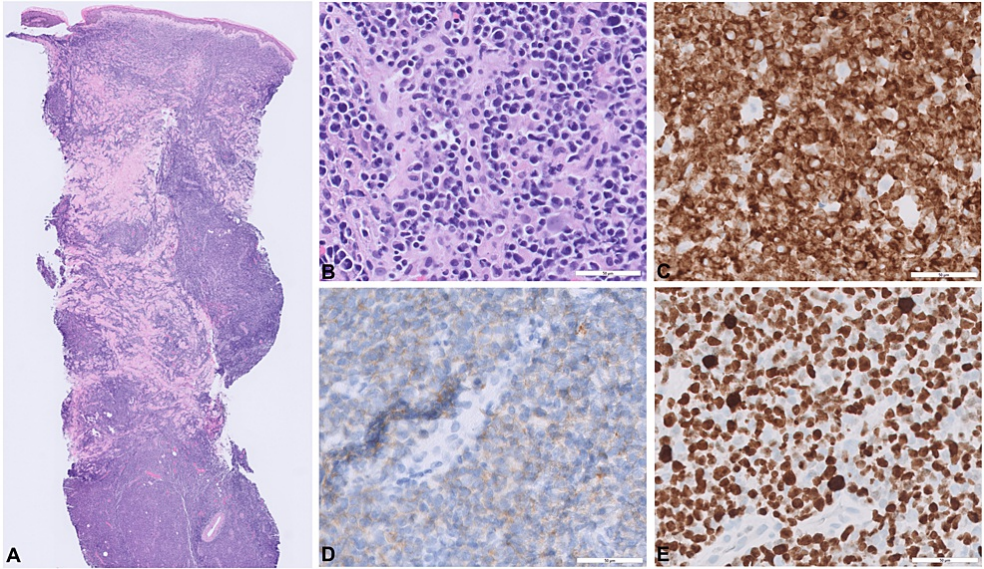
Biopsy of the left lower leg (A) Scanning power demonstrates a dense lymphoid infiltrate involving the reticular dermis and subcutaneous tissue (H&E, x 20). (B) Higher magnification shows medium to large atypical cells with irregular nuclei (H&E, x 400). Immunohistochemistry shows the neo-plastic cells diffusely positive for CD3 (C; x 400) and CD56 (D; x 400). (E) Ki-67 proliferation index is high (~90%) (Ki-67, x 400)

A punch biopsy was performed, which revealed an atypical lymphoid infiltrate with positive staining for CD2, CD3, CD30 (weakly), and CD56, and diffusely positive for Epstein-Barr encoding region (via in-situ hybridization), and Ki-67 staining of 80-90% of infiltrate (Figure 2). The histological and immunohistochemical features supported a diagnosis of ENKTL.

The patient was then referred to Oncology and Radiation Oncology for further evaluation and management. A PET scan did not reveal any evidence of nodal involvement or distant disease, and his condition was classified as stage IIE. The patient received concurrent chemoradiation for localized disease: 5580 cGy at 180 cGy per fraction for 31 fractions and DeVIC (dexamethasone, etoposide, ifosfamide, carboplatin) chemotherapy with a cycle length of 21 days for three cycles. Post-treatment PET scan showed complete remission of the disease.

**Figure 3:**
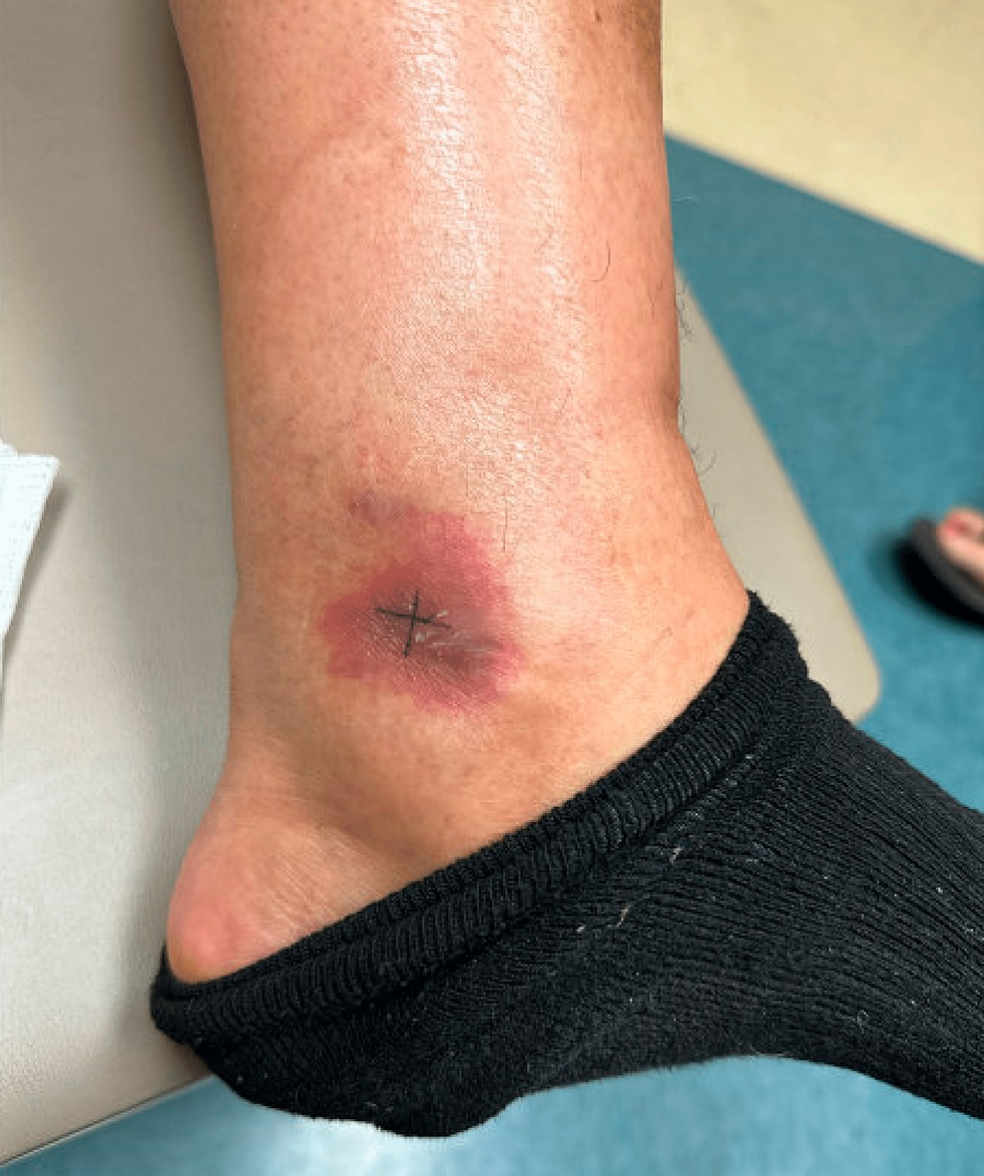
Recurrent extranodal natural killer/T-cell lymphoma (ENKTL) lesion above the left medial malleolus

**Figure 4:**
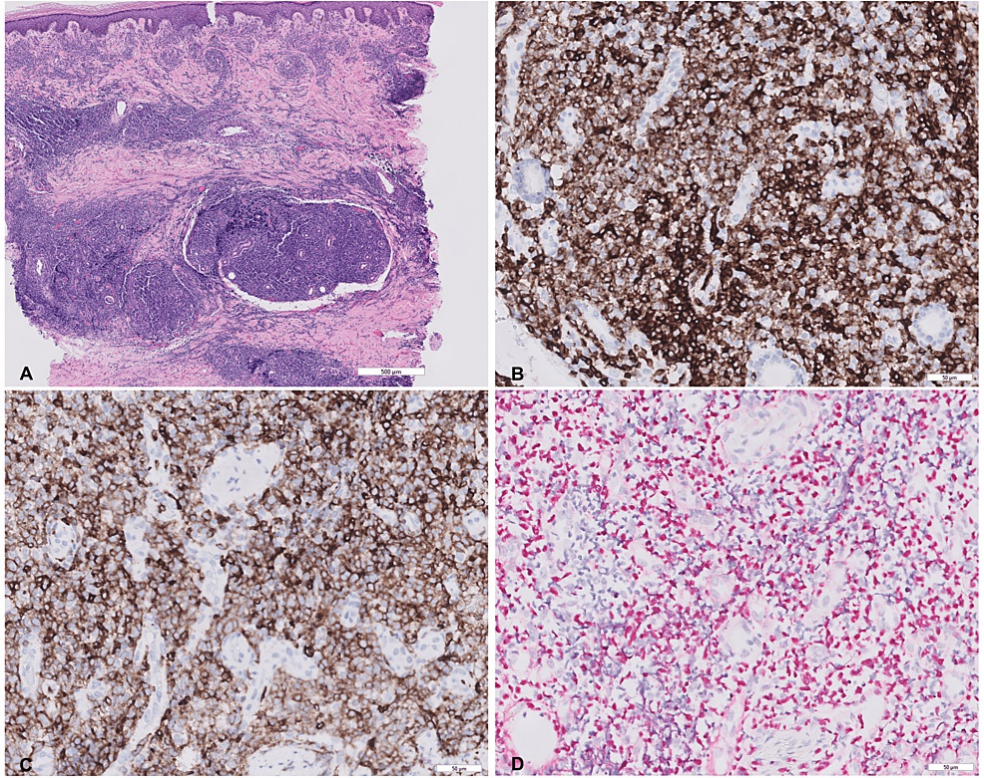
Biopsy of the left ankle (A) Dense lymphocytic infiltrate in the reticular dermis and subcutaneous tissue with prominent periadnexal and peri-vascular distribution (H&E, x 20). Immunohistochemistry shows the neoplastic cells diffusely positive for CD3 (B; x 200) and CD7 (C; x 200). (D) Epstein-Barr encoding region (EBER) in-situ hybridization is positive (EBER ISH, x 200)

Despite the initial favorable response, a new left ankle lesion appeared seven months after the completion of treatment. On examination, there was a violaceous plaque on the left medial ankle (Figure 3). PET scan showed mildly hypermetabolic skin thickening of the area with no evidence of distant disease. Biopsy from the ankle lesion showed findings similar to the initial biopsy, consistent with relapsed disease (Figure 4). The patient was restarted on chemoradiation with the VIPD regimen (etoposide, ifosfamide, cisplatin, dexamethasone).

## Discussion

ENKTL lesions may present as papules, subcutaneous nodules, erythema, and cellulitis-like or ulcerated plaques on the skin with a predilection for the extremities and trunk [1-4]. The disease predominantly affects middle-aged males with a higher incidence in Asian countries [5,6]. ENKTL has a poor prognosis with a median survival period of less than 12 months [5-8]. Median survival time for cutaneous ENKTL ranges from a few months up to about 15 months, and the condition accounts for about 10% of all ENKTL cases [2,4]. Epstein-Barr virus (EBV) has also been postulated to have a role in the pathogenesis and is nearly always present in ENKTL cases [6].

Histopathology typically demonstrates an angiocentric lymphoreticular infiltrate with deep tissue involvement [4-6,9]. Most commonly, NK cells are found in the dermis, while T-cells are rarely found [1]. The tumor causes vascular damage, leading to the characteristic necrotic clinical appearance [10]. Immunostaining classically reveals positive staining with cytotoxic marker(s), particularly CD2, cytoplasmic CD3, and/or CD56 positivity [5,11].

There are currently no standardized treatments for cutaneous ENKTL [2,3]. Despite a generally poor prognosis, advancements in the last few decades have considerably improved outcomes for patients with ENKTL [10]. Current treatment options include radiotherapy and/or chemotherapy [5]. Chemotherapy regimens include DeVIC, which has been noted to have a very favorable response in ENKTL; VIPD, which has been associated with improved outcomes when used after concurrent chemoradiation treatment; SMILE (steroid, methotrexate, ifosfamide, L-asparaginase, etoposide), which has been shown to be efficacious even for late-stage disease; and VIDL (etoposide, ifosfamide, dexamethasone, and L-asparaginase) [11,12-15]. Radiotherapy can be effective for localized disease but with rates of recurrence as high as 77% [5,6,9]. The metastatic disease typically requires concurrent chemotherapy and radiotherapy.

## Conclusions

Our patient initially underwent chemoradiation with a favorable response to the lesion in his left lower leg but subsequently had a recurrence of the disease on the left ankle several months later. He continues to be followed up by Oncology and Radiation Oncology with regular skin checks with Dermatology. Although ENKTL is very rare, especially in North America, dermatologists should be vigilant about possible cases as they may progress rapidly with aggressive behavior. Therefore, in patients with erythematous, nodular, cellulitis-like lesions refractory to antibiotics, a diagnosis of ENKTL should be considered.
